# Editorial: Gut microbial response to host metabolic phenotypes, volume II

**DOI:** 10.3389/fnut.2023.1136510

**Published:** 2023-02-03

**Authors:** Hui Han, Yong Su, Jie Yin

**Affiliations:** ^1^College of Animal Science and Technology, Nanjing Agricultural University, Nanjing, China; ^2^State Key Laboratory of Animal Nutrition, Institute of Animal Science, Chinese Academy of Agricultural Sciences, Beijing, China; ^3^College of Animal Science and Technology, Hunan Agricultural University, Changsha, China

**Keywords:** gut microbiota, microbial response, host, metabolism, phenotypes

Numerous studies have emphasized the importance of gut microbiota in modulating various physiological functions, including metabolism, inflammation, and neural development (1–3). Gut microbiota can not only affect the digestion and absorption of nutrients but also produce numerous metabolic bioactive signaling molecules to regulate host metabolism (4–6). A comprehensive understanding of the interaction of gut microbiota and host metabolism will create opportunities for new therapeutic approaches to the treatment of metabolic disorders.

It has been well-established that the gut microbiota is sensitive to dietary components, especially carbohydrates, fats, and proteins. In this Research Topic, dietary protein (Wang, Peng et al.) and starch (Wang, Zhou et al.) have been reported to shape microbial composition. Additionally, Hou et al. thoroughly discussed the role of gut microbiota in host metabolism, including carbohydrate, lipid, amino acid, and nucleic acid metabolism. These studies systematically interrogated the impact of diets with varying protein and starch content and illustrated the complex association between diets, gut microbiota and host metabolism. Moreover, studies have also revealed the diversity and characteristics of gut microbiota along the gastrointestinal tract (GIT) by using pig as a physiological relevant model of human metabolism. For example, Song et al. found that microbial richness and diversity gradually increased from the small to large intestine. Moreover, the bacterial composition was different between the small and large intestine, which might due to differing physiological functions as required by the host. Like bacteria, gut fungi is also an important part of the intestinal microbiota that interacts with host metabolism (7). However, studies on characterizing the gut fungal diversity and composition along the whole GIT are limited. In this Research Topic, Li et al. reported that the difference in the gut fungal diversity and composition along the GIT sections was smaller than that between batches in pigs.

Alterations of gut microbiota have been implicated in the pathogenesis of metabolic disorders. In this Research Topic, Dong et al. used metagenomic and metabolic methods to investigate the changes in gut microbiota (including bacteria, bacteriophage, and archaea) in mice with obesity and atherosclerosis. Long et al. gave a comprehensive overview regarding the association between gut microbiota-derived metabolites and pathogenesis of ischemic stroke. Building on the complex association between gut microbiota and metabolic disorders, studies have aimed to evaluate the causality of gut microbiota in host metabolism by using antibiotics and fecal microbiota transplantation (FMT). However, the effectiveness and impacts of FMT on specific bacterial strains remain unclear. Tan et al. showed that an antibiotic cocktail containing vancomycin, ampicillin, neomycin, and metronidazole in drinking water effectively eliminated the microbial strains belonging to *Bacteroidets, Actinobacteria*, and *Verrucoomicrobia*, which can be restored by transplantation of microbiota from healthy control mice.

Laboratory and clinical studies have highlighted the potential to use interventions related to gut microbiota for treating metabolic disorders (8–10). For example, probiotics containing either single or multiple microorganism strains (especially strains of genera *Lactobacilli* and *Bifidobacteria*) have proven to be effective therapeutic approaches for alleviating metabolic disorders (11–13). Similarly, in this Research Topic, studies showed that some strains of *Lactobacillus* applied in asthma (Wang W. et al.) and obesity (Ma Y. et al.) were able to modify the gut microbial composition and exhibit beneficial effects on host. Additionally, Guo et al. found that dietary intervention with oropharyngeal probiotic ENT-K12 effectively reduced episodes of upper respiratory tract infections in children with recurrent respiratory tract infections during high peak season. Besides, dietary prebiotics have been demonstrated to alter gut microbiota and impart favorable metabolic benefits. In this respect, studies in this Research Topic reported that extracts from bearberry (Ma J. et al.), dark tea (Wang C. et al.), Artemisia argyi leaf (Wang, Ma et al.), Aurantii Fructus Immaturus (Chen et al.), and cannabis (Gong et al.) were able to positively alter gut microbiota and improve metabolism and inflammation. These studies may provide novel therapeutic strategies for treating metabolic diseases.

Overall, studies in this Research Topic promoted the understating of the role of gut microbiota in host metabolism, although the precise mechanism was not fully clear. Furthermore, data from the above mentioned studies offered microbiome-based strategies for alleviating metabolic diseases. However, additional studies are necessary to further shed light on the complex interaction between gut microbiota and host metabolism in order to find opportunities to alleviate metabolism-related diseases. Additionally, the evidence of concept generated in animal models need to be further translated to clinical setting.

## Author contributions

All authors listed have made a substantial, direct, and intellectual contribution to the work and approved it for publication.
